# Metabolic modulation of immune cell function: mechanisms and therapeutic implications in cancer immunotherapy

**DOI:** 10.1038/s41389-026-00622-4

**Published:** 2026-05-07

**Authors:** Nina Fenouille, Camille Lobry, Lina Benajiba, Alexandre Puissant

**Affiliations:** 1 INSERM UMR 1342, Institut de Recherche Saint Louis, Institut de la Leucémie Paris-Saint Louis, Université Paris Cité, Paris, France; 2 APHP, Hôpital Saint-Louis, Centre d’Investigations Cliniques, INSERM, CIC 1427, Université Paris Cité, Paris, France

**Keywords:** Immunosurveillance, Cancer microenvironment, Metabolism

## Abstract

Immune cell function is remarkably plastic, allowing T cells, NK cells, and macrophages to transition from resting or quiescent states to proliferative, cytotoxic, or inflammatory programs. These functional shifts are tightly coupled to metabolic reprogramming, which not only fuels energy and biosynthesis but also shapes epigenetic and transcriptional landscapes that guide immune responses. In this review, we highlight how intrinsic metabolic pathways which include glycolysis, fatty acid oxidation, amino acid metabolism, and TCA cycle intermediates, regulate T and NK cell proliferation, cytotoxicity, memory formation, and epigenetic programs. We also examine macrophages, whose polarization into pro-inflammatory M1 or tissue-reparative M2 states is orchestrated by distinct metabolic programs such as arginine metabolism, oxidative phosphorylation, and fatty acid oxidation, with consequences for local immune regulation. We then explore how tumors exploit these metabolic dependencies to create hostile microenvironments that restrict nutrients, accumulate immunosuppressive metabolites, and dampen immune cell activity. Finally, we discuss emerging metabolic interventions designed to restore immune fitness, enhance the efficacy of immune checkpoint inhibitors, and improve the persistence and cytotoxicity of adoptive T cell therapies, including CAR-T cells, in nutrient-deprived and hypoxic tumor niches. By linking immune cell plasticity to metabolic control, this review provides a framework for understanding how metabolism shapes immunity and identifies strategies to harness these pathways for next-generation cancer immunotherapies.

## Introduction

Metabolism plays a critical role in the maturation and effector functions of both myeloid and lymphoid immune cell subsets. Among these, T cells, natural killer (NK) cells, and macrophages have emerged as central players in cancer immunotherapy, each relying on distinct yet complementary mechanisms to counter tumor immune evasion. These immune populations act in a coordinated manner to shape antitumor immunity: macrophages regulate antigen presentation and inflammatory cues, NK cells provide rapid, antigen-independent cytotoxic responses, and T cells mediate antigen-specific and long-term immune control. Crosstalk among these immune cell types, through cytokines, cell-cell interactions, and metabolic signaling, is therefore essential for effective tumor surveillance and optimal therapeutic outcomes.

T cell-based approaches, such as immune checkpoint blockade (i.e., PD1/PD-L1- and CTLA-4-directed monoclonal antibodies) and adoptive cell therapies like chimeric antigen receptor (CAR) T cells, have demonstrated remarkable clinical success, particularly in hematologic malignancies, for which several of these therapies are now FDA-approved. NK cell-based therapies, like adoptive NK cell transfer, CAR-NK cells, and NK cell-engaging antibodies, provide potent anticancer activity without requiring prior antigen sensitization and generally exhibit lower overall toxicity compared to T cell-based therapy systems. Macrophages, which are abundant in the tumor microenvironment (TME), are being therapeutically reprogrammed from tumor-promoting M2-like phenotypes toward pro-inflammatory M1-like states or engineered as CAR-macrophages to enhance tumor clearance and remodel the immunosuppressive TME.

Recent advances in immunometabolism have refined our understanding of the metabolic cues that govern immune cell development and function, as well as how tumors exploit metabolic pathways to impair antitumor immunity. Building on this knowledge, metabolic modulation strategies, such as nutrient supplementation, inhibition of specific metabolic enzymes, and dietary interventions, are now explored to optimize existing immunotherapeutic approaches and convert immunosuppressive tumor niches into immune-responsive environments. This integrated approach is advancing rapidly, positioning immunometabolism at the forefront of cancer therapy.

In this review, we provide an overview of how metabolism regulates the development and function of T cells, NK cells, and macrophages, either directly through intrinsic metabolic pathways or indirectly through epigenetic and transcriptomic remodeling. We also discuss how tumors exploit these metabolic vulnerabilities to create an immune-hostile microenvironment that compromises immune cell activity. Finally, we highlight recent metabolic interventions aimed at suppressing tumor-derived immunosuppressive signals, enhancing the efficacy of immune checkpoint inhibitors (ICIs), and improving the persistence and cytotoxicity of adoptive T cell therapies, offering promising avenues to optimize the antitumor effects of immunotherapies.

## Metabolic regulation of T and NK cells

T lymphocytes and NK cells undergo extensive metabolic reprogramming to adapt to diverse environmental stressors encountered during development and upon interaction with target tissues or cells. Numerous studies have highlighted critical roles for specific metabolic pathways and metabolites in guiding maturation, modulating lymphocyte signaling, determining functional outcomes and cell fate, and shaping their ability to engage target cells. These metabolic pathways can influence T and NK cell function and signaling directly or indirectly by remodeling their epigenetic landscape, thereby altering their behavior.

### Direct metabolic regulation of T and NK cell development and function

#### Metabolic regulation of T cell development

In the thymus, early T cell development is characterized by distinct metabolic changes that shape the multiple checkpoints and proliferative steps leading to the maturation of naive T cells. Early thymic progenitors (DN1) differentiate into DN2 thymocytes, committing to the T-cell lineage, and advance to the DN3 stage, where β-selection ensures functional pre-TCR assembly [1, 2]. Successfully selected thymocytes progress to the DN4 stage, undergo rapid proliferation driven by complex metabolic regulation, and diverge into either γδ or αβ T cell lineages. They subsequently enter a quiescent double-positive (DP) stage, where positive and negative selections occur, before maturing into single-positive (SP) CD4+ or CD8+ T cells.

**Fig. 1:**
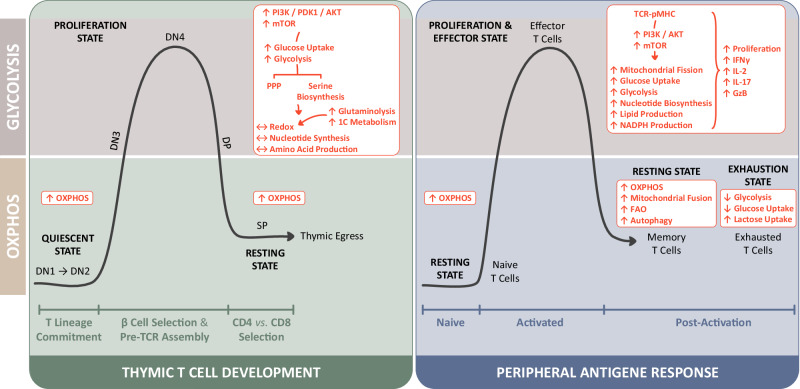
OXPHOS-to-glycolysis metabolic transition in T cells. Both thymic development and peripheral antigen responses follow a conserved metabolic trajectory: quiescent DN1→DN2 thymocytes and naïve T cells rely on mitochondrial oxidative phosphorylation (OXPHOS), proliferating DN3→DP thymocytes and antigen-activated T cells shift to glycolysis, and their return to OXPHOS marks CD4/CD8 selection, thymic egress, or memory T cell formation. These transitions are coupled to the rewiring of key signaling and metabolic pathways (red rectangles). PPP pentose phosphate pathway, FAO fatty acid oxidation.

Consecutive waves of selection and proliferation at each stage of thymocyte maturation drive profound metabolic changes. From DN1 to DN3, thymocytes shift from a metabolically quiescent state relying primarily on oxidative phosphorylation (OXPHOS) to a highly proliferative, glycolysis-dependent state [3] (Fig. 1). Consistent with this observation, independent studies showed that impairing the glycolytic genes *Glut1* and *Pkm2* disrupts the DN-to-DP thymocyte transition [4, 5]. The DN3 phase marks the onset of robust proliferation, during which thymocytes undergo V(D)J rearrangement and β-selection, both of which are essential for proper pre-TCR assembly and subsequent T cell development. Pre-TCR, IL7, and NOTCH signaling converge to activate pathways such as PI3K/PDK1/AKT and mTOR, facilitating the glycolytic switch that supports the proliferative burst following β-selection [6–8]. Knocking out the mTOR-containing complex, mTORC1, in T cells affects their lineage commitment [9]. The energetic switch toward aerobic glycolysis helps thymocytes meet the biosynthetic requirements for cell growth and division [10]. Intermediates from glucose catabolism during glycolysis feed into the pentose phosphate and serine biosynthesis pathways, supporting nucleotide and amino acid production [11]. Concomitant increases in glutaminolysis and one-carbon metabolism further sustain nucleotide and amino acid synthesis while maintaining redox balance during continuous proliferation [12]. Following this critical proliferative phase, T cells return to a resting state characterized by a shift back to an OXPHOS-dominated metabolism as they undergo positive and negative selection.

#### Metabolic regulation of T cell function

T cells that pass positive and negative selection mature into CD4+ helper and CD8+ cytotoxic T cells, exit the thymus, and populate peripheral lymphoid organs such as lymph nodes and spleen, where they remain quiescent until antigen encounter. Upon recognition of antigen-MHC complexes and co-stimulatory signals such as CD28, they rapidly proliferate and differentiate under the influence of cytokines such as IL2. The cytokine milieu and local environment shape their fate, driving CD8+ cells toward cytotoxic activity and CD4+ cells into various subsets, which include Th1, Th2, Th17, or regulatory T cells (Treg). After antigen clearance, some effector T cells transition into memory cells, enabling rapid responses upon re-exposure. This remarkable plasticity of T cell responses is tightly regulated by complex signaling pathways and profound metabolic reprogramming, which are dynamically tailored to the functional needs of distinct T cell subsets, each exhibiting unique metabolic features [13].

##### An OXPHOS-to-glycolysis switch supports transition in T cell activity

One key metabolic feature of T cell biology is the **energetic switch** that occurs during their transition from naive to activated states and, later, to memory or exhausted states. This transition mirrors the OXPHOS-to-glycolysis-to-OXPHOS metabolic pattern observed during early thymic T cell development (Fig. 1). Like DN1 thymocytes, naive T cells primarily rely on OXPHOS and require minimal nutrient intake to maintain homeostasis [14].

Upon activation, effector T cells shift toward anabolic metabolism dependent on glycolysis and glutaminolysis, similar to thymocytes undergoing β-selection [15]. This switch is driven by TCR-induced PI3K/AKT/mTOR signaling, which promotes mitochondrial fission, reduces respiratory capacity, upregulates GLUT1, and induces glycolytic regulators such as MYC and HIF1A [16–21]. In contrast, impaired TCR signaling diminishes glycolysis and anabolic metabolism [22]. Enhanced glycolysis supports effector T-cell proliferation and effector molecule production, such as IFNγ, IL2, IL17, GZMB, by providing metabolic intermediates such as ribose sugars for nucleotide synthesis, glycerol and citrate for lipid biosynthesis, nonessential amino acids, and NADPH via the oxidative pentose phosphate pathway [15, 23–25]. Glycolytic mediators can also directly regulate T-cell function; for instance, in the absence of glycolysis, the glycolytic enzyme Gapdh binds to and suppresses *Ifng* mRNA translation [15], whereas the glycolytic intermediate phosphoenolpyruvate promotes calcium signaling essential for T-cell activation [26]. Moreover, enhanced ATP production through glycolysis reinforces PI3K signaling by sustaining strong PI3K activity, thereby establishing a positive feedback loop in which PI3K activation drives the metabolic program which is required to maintain its own signaling [27].

After antigen clearance, memory T cells persist in circulation, enabling rapid recall responses to subsequent antigen exposure [28]. These memory T cells revert to a metabolic program similar to naive T cells, relying on OXPHOS fueled at least in part by fatty acid oxidation (FAO), and autophagy [29]. Compared with effector T cells, memory T cells have greater mitochondrial mass, fused mitochondrial ultrastructure, and higher spare respiratory capacity, features that are presumably important to support their long-term survival in the absence of antigen [19]. Reactive oxygen species (ROS) generated by mitochondrial complex III play a critical role in memory CD8+ T cell generation, as genetic abrogation of this component of ROS production, while maintaining normal OXPHOS capacity, results in the early formation of memory precursor CD8+ T cells that are subsequently lost at later time points after antigen stimulation [30]. mTOR pathway is also an important regulator of memory T cell differentiation, as both pharmacological and genetic inhibition of the mTORC1 complex accelerate the formation of memory T cells [31], at least in part through activation of SREBP [20, 32]. In addition to its role in memory formation, mTOR signaling exerts distinct and reciprocal effects on CD4+ T helper cell differentiation: mTORC1 signaling promotes Th1, Th2, and Th17 differentiation, whereas mTORC2 signaling—defined by the interaction of the core kinase mTOR with Rictor rather than Raptor, the defining subunit of mTORC1—favors Th2 differentiation [20, 33]. Consistent with this, *Rictor*-deficient T cells fail to differentiate into Th2 cells [34]. Divergent mTOR signaling outputs in CD4+ T cell differentiation may reflect distinct strategies of mTORC1 inhibition: suppression of the structural scaffolding subunit *Raptor* was used in a first study [20], whereas knockout of the upstream kinase activator *Rheb* was employed in the latter [33] to impair mTORC1 signaling.

Chronic antigen exposure, as seen in persistent viral infections or cancer, or insufficient CD4+ T cell and cytokine support, drives CD8+ T cells into a terminally differentiated state known as T cell exhaustion. Two main subsets were initially described: progenitor exhausted T cells (Tpex) and terminally exhausted T cells (Tex) [35]. Tpex cells display intermediate PD-1 expression, retain self-renewal via TCF-1, and can differentiate further under sustained TCR stimulation into Tex cells, which lose self-renewal, produce fewer effector cytokines (IL2, TNFα), and upregulate inhibitory receptors (PD-1, CTLA-4, TIM-3, LAG-3) [36]. Throughout chronic infection, Tpex cells retain their metabolic characteristics and the ability to activate mTOR signaling in response to TCR stimulation, whereas Tex cells undergo metabolic exhaustion, including a loss of mTOR signaling [37]. Early during exhaustion, elevated PD-1 signaling impairs mitochondrial function and reduces glucose uptake and glycolysis, at least in part through repression of the metabolic regulator PGC-1α [38]. Consistent with this, in vitro engagement of PD-1 or CTLA-4 suppresses glycolysis in activated T cells [39]. Additionally, Tex cells exhibit mitochondrial dysfunction following chronic antigen exposure, which leads to a reduced NAD^+^/NADH ratio and increased ROS production rather than the maintenance of cellular nucleotide triphosphate levels [40]. Tex cells also upregulate the lactate transporter MCT11 (SLC16A11), which increases lactate influx, thereby limiting T cell effector function, a defect that can be reversed by conditional *MCT11* deletion [41]. More recently, single-cell-based approaches have allowed a deeper understanding of the gene regulatory networks driving CD8+ T-cell differentiation toward terminal exhaustion. Large-scale in vivo single-cell CRISPR screens mapped the fate regulome of tumor-infiltrating CD8+ T cells and thereby revealed a detailed progression from Tpex cells to proliferative intermediate Tex1 cells and finally to terminally exhausted Tex2 cells [42]. The transcription factors IKAROS/IKZF1 and ETS1 orchestrate successive steps in this process: IKAROS promotes metabolic and mTORC1-related gene programs in Tpex1 cells, driving their differentiation into Tpex2 cells, while ETS1 acts as a gatekeeper for the Tpex2-to-Tex1 transition, likely by restraining mTORC1 activity and metabolic reprogramming.

##### Cholesterol metabolism influences TCR activation

A second key metabolic feature of T cell function involves **lipid metabolism**, which critically influences TCR activation [43]. Early studies focused on lipid rafts—plasma membrane microdomains enriched in specific lipids and proteins—that facilitate TCR signaling by clustering components such as the Src-family kinase, LCK [44]. Cholesterol also emerged as a major TCR regulator because this lipid is capable of direct binding to the TCRβ transmembrane domain to either stabilize the inactive TCR and prevent activation in the absence of antigen-MHC complex, or promote TCR nanocluster formation, thereby enhancing antigen sensitivity [45–47]. Inhibiting ACAT1, the key cholesterol esterification enzyme, enhances CD8+ T-cell proliferation and effector function by increasing plasma membrane cholesterol, which promotes TCR clustering, signaling, and immunological synapse formation [48]. In contrast, cholesterol sulfate, a naturally occurring form of cholesterol, inhibits early TCR signaling by disrupting TCR-CD3 nanoclusters, likely through interference with cholesterol binding, thereby impairing downstream signal transduction [49]. In addition, lysophosphatidic acid, a bioactive phospholipid derived from phosphatidylcholine metabolism, disrupts immune synapse formation by impairing TCR-induced actin and microtubule remodeling and downstream signaling pathways [50, 51].

##### Amino acid metabolism and uptake fuels T cell activation and effector functions

TCR engagement and co-stimulation also influence T cell metabolism by modulating amino acid pathway activity and uptake, which in turn shape T cell function. Although T cells can synthesize all the non-essential amino acids, they are auxotrophic for several of them and thus depend on external sources to support robust activation. This dependency on exogenous amino acids serves not only to fuel T cell activation and effector functions but also acts as a regulatory checkpoint. By limiting amino acid availability either through competition, enzymatic depletion, or amino acid transporter regulation, the immune system can temper T cell response, control tissue damage, and fine-tune immunity.

**Glutamine** is a non-essential amino acid that is nevertheless an obligate requirement for effective T cell differentiation, function, and proliferation. Glutamine is uniquely essential because its function cannot be compensated for by other metabolic precursors or downstream products [52]. Glutamine serves as both a bioenergetic and biosynthetic substrate for effector T cells. For example, during pathogen infection, CD8+ T cells channel glutamine into the TCA cycle to support oxidative phosphorylation and anabolic pathways, including aspartate synthesis. This glutamine-dependent aspartate production is critical for effector cell expansion and function, as silencing *Got1* impairs CD8+ T cell proliferation and IFNγ production in vivo [53].

Upon TCR engagement, glutamine uptake is enhanced through upregulation of the transporter SLC1A5 (ASCT2) [54], along with increased activity of glutamine-metabolizing enzymes such as GOT1/2 and GLUD1 [52]. In parallel, TCR activation induces a MYC-driven transcriptional program downstream of AKT and mTOR signaling that couples glutamine catabolism to additional biosynthetic pathways, including hexosamines, pyrimidines, purines, and polyamines [17]. Beyond effector differentiation, glutamine metabolism also plays a critical role in T cell memory formation, as knockdown of the glutamine transporter *Slc38a2* impairs memory T cell generation [55].

Importantly, modulation of glutamine metabolism at distinct enzymatic steps leads to markedly different CD4+ T-cell differentiation outcomes. Whereas glutamine deprivation or deficiency of the glutamine transporter *ASCT2* skews CD4+ T cells toward a Treg-like phenotype at the expense of Th1 and Th17 differentiation [54, 56], impairment of the first step of glutaminolysis through *GLS* deficiency selectively promotes Th1 differentiation and inhibits Th17 differentiation without affecting Treg cell generation [57].

CD8+ T cells are auxotrophic for **asparagine**, especially during early activation, due to low expression of asparagine synthetase, *ASNS*, which converts aspartate to asparagine, making them reliant on extracellular asparagine for proper activation [58, 59]. Extracellular asparagine restriction depletes intracellular asparagine and suppresses CD8+ T cell activation. However, this strict asparagine dependence diminishes over time, as prolonged stimulation induces ASNS upregulation [58]. Cells switch to de novo asparagine biosynthesis to replenish the intracellular asparagine pool, reducing overall carbon consumption and disposal and enhancing nucleotide biosynthesis [60]. The essential role of asparagine metabolism in T cells underlies its therapeutic targeting in T-cell acute lymphoblastic leukemia (T-ALL), a malignancy caused by uncontrolled hyperproliferation of immature T lymphocytes. Administration of asparaginases now constitutes the backbone of treatment for T-ALL during the induction phase of chemotherapy to deplete asparagine and impair leukemic cell survival.

**Arginine** is another key non-essential amino acid component of T cell function. Arginine starvation or silencing of its transporter expressed in human T cells, *SLC7A1*, leads to downregulation of the CD3ζ chain, reduced T cell proliferation, and decreased cytokine production [61, 62]. In contrast, arginine supplementation promotes the generation of central memory-like T cells with enhanced survival capacity, thereby improving CD8+ T cell anti-tumor activity [63]. Finally, **serine** is another non-essential amino acid that can be supplied either through de novo synthesis from glucose or via uptake from the extracellular environment, both of which are required to support optimal T cell proliferation [64, 65].

Among the essential amino acids, several have been reported as critical regulators of T cell proliferation and function. **Tryptophan** metabolism plays an important role in modulating T cell responses. Tryptophan is catabolized into kynurenine by the immunoregulatory enzyme indoleamine 2,3-dioxygenase (IDO1), which is preferentially expressed in macrophages and dendritic cells [66]. Kynurenine produced by these cells acts in a paracrine manner to negatively regulate T cell activity [67]. Although T cells do not constitutively express IDO1, they import kynurenine primarily through active transport via SLC7A5 [68]. Once inside T cells, kynurenine directly activates the aryl hydrocarbon receptor (AhR). Upon ligand binding, AhR translocates from the cytosol to the nucleus, where it binds to response elements in target gene promoters. Kynurenine-mediated AhR activation drives immunosuppressive programs by inducing CD8+ T cell death, upregulating PD-1 expression, and promoting Treg differentiation [69]. In contrast, glutarate, another metabolite derived from tryptophan and lysine catabolism, positively modulates CD8+ T cell function. It regulates the pyruvate dehydrogenase complex (PDHc) through post-translational glutarylation. Supplementation with the glutarate precursor diethyl glutarate decreases PDH activity, improves mitochondrial function, and enhances CD8+ T cell cytotoxicity [70].

Essential amino acids include the branched-chain amino acids (BCAAs) — **leucine,** **isoleucine**, and **valine** — which must be obtained from the diet. They contribute to de novo protein synthesis and structural maintenance and serve as precursors for sterol and keto-body biosynthesis. Accumulation of intracellular leucine via the SLC7A5 transporter activates the mTOR signaling pathway, leading to hyperactivation of CD4+ T cells [71]. In addition, TCR activation in CD8+ T cells induces upregulation of the BCAT1 enzyme, which catalyzes the first step of BCAA breakdown. Consistent with this, BCAA supplementation promotes glucose uptake and enhances CD8⁺ T cell activity, increasing cytokine production and cytotoxicity [72].

##### Metal ions are important regulators of T cell signaling

Metal ions, which include zinc (Zn²⁺), manganese (Mn²⁺), magnesium (Mg²⁺), potassium (K⁺), calcium (Ca²⁺), iron (Fe²⁺/Fe³⁺), and copper (Cu⁺/Cu²⁺), are key components of metabolism and important regulators of T cell function, owing to their roles in promoting various intracellular signaling cascades that control immunological memory and immune responses. **Calcium** and **magnesium** are the most abundant metal ions involved in TCR signaling. Upon TCR engagement, calcium stored in the endoplasmic reticulum (ER) is released, triggering a conformational change in the ER calcium sensor STIM1. Activated STIM1 migrates to ER-plasma membrane junctions, where it binds to the ion channel ORAI1, opening a pore that allows a rapid influx of extracellular calcium into the cytosol. This Ca²⁺ influx also activates the potassium channels Kv1.3 and KCa3.1, promoting potassium efflux to restore membrane potential and sustain calcium entry [73]. This process, known as store-operated Ca²⁺ entry (SOCE), is the primary mode of calcium influx in T cells. In parallel, Mg²⁺ influx occurs in a MAGT1-dependent manner [74]. Importantly, extracellular methionine is critical to regulate Ca^2+^ influx through modulation of the methylation level of KCa3.1 [75].

The coordinated entry of calcium and magnesium activates the NFAT pathway, driving the T cell activation program, which includes polarization, expression of activation markers such as CD25 and CD69, proliferation, migration, and secretion of IL2, IFNγ, TNFα, perforin, and granzymes [76, 77]. Calcium further promotes glycolysis and oxidative phosphorylation, both of which are essential, as noted above, for optimal T-cell activation [78]. Notably, the ER is not the only organelle involved in Ca²⁺ influx. Following immune synapse formation upon TCR-MHC interaction, mitochondria translocate to the vicinity of the synapse, enabling a larger and more sustained calcium influx and concomitant NFAT activation [79]. In addition, mitochondrial ROS also contributes to promoting NFAT activation [80]. Mg²⁺ further modulates T cell activation through T cell co-stimulation molecules, especially the integrin LFA-1, whose function depends on extracellular magnesium. By sensing extracellular Mg²⁺ levels, LFA-1 regulates Ca²⁺ influx and stabilizes immune synapse formation, thereby fine-tuning the magnitude and quality of CD8+ T cell response [81].

During antigen-driven T-cell responses, **zinc** and **copper** also act as important modulators of immune function. Zinc regulates key signaling pathways such as MAPK, PI3K/AKT, and STAT3, shaping gene expression programs that control T-cell differentiation, survival, proliferation, and apoptosis [82]. Copper similarly supports cytotoxic T lymphocyte killing capacity, promotes T-cell proliferation in vitro and in vivo, providing an additional layer of regulation over T-cell activity [83].

**Iron** is another major metal ion with pleiotropic effects essential for both CD4+ and CD8+ T cell activity. Defective transferrin receptor function or reduced intracellular Fe²⁺ levels impair T cell activation, as evidenced by diminished CD25 expression and defective IL2 receptor signaling [84]. Iron also promotes IL2 production in CD4+ T cells by preventing the proteolysis of the RNA-binding protein PCBP1 [85]. In CD8+ T cells, iron supports mitochondrial integrity and TCA cycle activity, both of which are critical for meeting the metabolic demands of T cell activation [86].

**Fig. 2:**
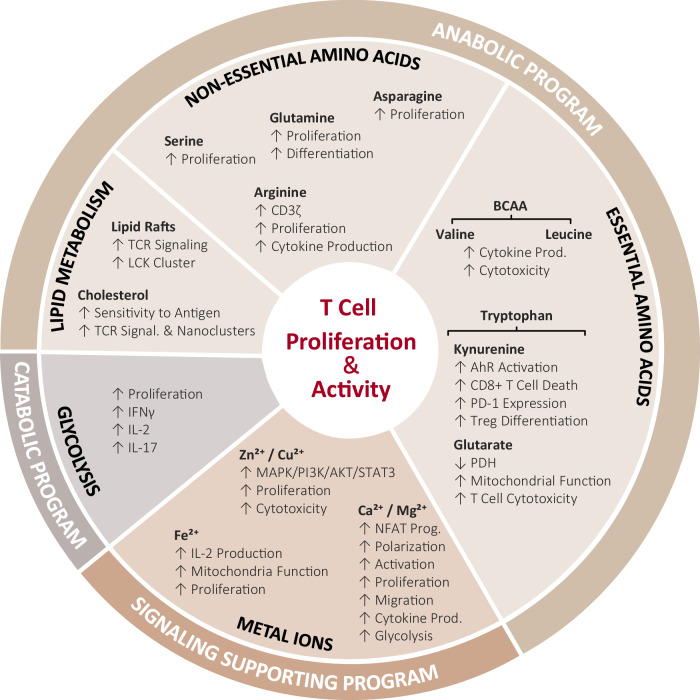
Metabolic rewiring sustains T cell proliferation and function. Both catabolic and anabolic pathways—including glycolysis, lipid metabolism, amino acid metabolism (essential and non-essential), and metal ion metabolism—support key T cell functions. These metabolic programs provide the necessary building blocks for proliferation, sustain effector functions through the production of cytokines and effector molecules, and enable proper T cell signaling.

In summary, proper T lymphocyte development and function rely on the coordinated engagement of multiple metabolic programs that act at key nodes of T cell activation and proliferation. These include dynamic shifts between catabolic pathways such as glycolysis and oxidative phosphorylation during transitions between resting and proliferative states, activation of anabolic programs dependent on amino acid uptake and lipid metabolism, and transient bursts of metal ion uptake to sustain critical signaling cascades downstream of TCR engagement (Fig. 2). Of note, although CD8+ T cells primarily utilize conventional fuels such as glucose and glutamine, they exhibit remarkable **metabolic flexibility** and can also exploit other physiological carbon sources and nutrients, such as lactate, when available at sufficient concentrations (e.g., >100 μM in mouse serum) to support TCA cycle metabolism and biomass generation even in the presence of glucose [87]. This adaptability occurs independently of transcriptional changes, thereby indicating that activated T cells inherently adjust fuel utilization based on nutrient availability. When these external carbon sources are abundant, they are preferentially oxidized alongside glucose, reducing glucose’s contribution to the TCA cycle. Importantly, the presence of abundant external carbon sources enhances T cell viability and effector molecule production, underscoring how environmental nutrient availability and fuel choice critically shape T cell function and survival in diverse immune microenvironments [87].

#### Metabolic regulation of NK cell function

NK cells are cytotoxic innate lymphocytes that bridge innate and adaptive immunity by providing rapid defense against viral infections and malignant transformation. Unlike T and B cells, NK cells do not require receptor gene rearrangement, allowing them to respond immediately to stress signals by killing target cells, secreting IFNγ, and shaping T cell responses depending on the context [88]. Human NK cells are commonly divided into two subsets on the basis of CD56 expression. CD56^dim^ NK cells, which constitute the majority in peripheral blood, are specialized for immediate cytotoxicity, whereas CD56^bright^ NK cells display greater metabolic flexibility and immunoregulatory capacity, requiring upregulation of cytotoxic machinery before achieving full effector potential. Consistent with these functional distinctions, cytokine stimulation induces stronger metabolic responses in CD56^bright^ than in CD56^dim^ cells and promotes higher expression of nutrient transporters such as *GLUT1*, *SLC1A5*, *SLC7A5*/*SLC3A2*, and the intracellular iron transporter, *CD71* [89].

##### Functional impact of glycolysis and OXPHOS on NK cell function

At steady state, NK cells are metabolically quiescent, relying on low basal levels of **glycolysis** and **OXPHOS** [90]. However, their metabolic requirements shift dramatically upon activation. Short-term stimulation with IL12, IL15, or IL18 can trigger IFNγ production without substantially increasing glycolytic or mitochondrial flux [91, 92]. In contrast, prolonged cytokine exposure drives a profound metabolic reprogramming characterized by enhanced glucose uptake, induction of glycolytic enzymes, expansion of mitochondrial mass, and elevated glycolysis and OXPHOS [91, 92]. Interestingly, instead of engaging the canonical TCA cycle, activated NK cells preferentially utilize the citrate/malate shuttle to link glycolysis to respiration. This pathway exports citrate from mitochondria to the cytosol, where it is converted to acetyl-CoA for lipid biosynthesis and protein acetylation, and to malate for re-import into mitochondria to sustain electron transport chain activity [92]. Unlike many lymphocytes, NK cells can maintain OXPHOS even under glutamine deprivation or glutaminase inhibition, further emphasizing their unique metabolic wiring [93].

##### Serine, leucine, and arginine metabolisms are key regulators of NK cell function

Beyond glycolysis, amino acid metabolism critically shapes NK cell effector function. **Serine** metabolism is a central node, as exogenous serine fuels sphingolipid biosynthesis through serine palmitoyltransferase, generating sphingomyelin for membrane remodeling and sphingosine-1-phosphate for signaling. Human NK cells are particularly vulnerable to serine deprivation because they cannot perform de novo serine synthesis upon activation, leading to impaired cytotoxicity under dietary or tumor-driven serine restriction. In contrast, murine NK cells display metabolic flexibility, producing serine de novo to support one-carbon metabolism and proliferation. Nevertheless, in both species one-carbon metabolism and glutathione synthesis are indispensable for NK proliferation, IFNγ production, and the fine-tuning of cytotoxic versus inflammatory responses [94]. In addition, like in T cells, essential amino acids such as **leucine** and **arginine** promote proliferation and differentiation by activating mTOR-dependent signaling cascades, which also enhance TNFα and IFNγ production [95, 96].

##### Signaling and transcriptional control of NK cell metabolism

At the center of NK cell metabolic reprogramming lies **mTORC1**, which integrates cytokine and nutrient cues to drive effector outputs. Cytokine stimulation of resting NK cells activates mTORC1, leading to the upregulation of nutrient transporters, glycolytic enzymes, and mitochondrial biogenesis, while also directly supporting the production of IFNγ and granzyme B [91, 97]. Several transcriptional regulators acting downstream or in parallel to mTORC1 further promote NK cell metabolic rewiring. **SREBP** coordinates lipid biosynthesis while simultaneously enhancing glycolysis and OXPHOS through the citrate/malate shuttle; its genetic or pharmacological inhibition impairs NK cell metabolic fitness, reduces IFNγ and granzyme B production, and weakens antitumor responses [92].

**MYC** is another central regulator of the metabolic changes that support NK cell function. Upon activation with IL-2/IL-12, *MYC* upregulation is driven downstream of mTORC1 by SLC7A5 and glutamine. Together, MYC and SREBP represent two key factors in shaping the metabolic responses of IL-2/IL-12-stimulated NK cells: MYC primarily drives glycolysis and mitogenesis, whereas SREBP directs the metabolic switch toward the citrate/malate shuttle to fuel OXPHOS [93]. In addition, MYC cooperates with SREBP to promote polyamine biosynthesis via upregulation of the ornithine decarboxylase *ODC1*, a pathway that further supports glycolysis, OXPHOS, and subsequent IFNγ production [98].

More recently, **MEF2C** has been identified as an upstream regulator of SREBP signaling, linking PI3K/AKT/mTORC1 activity to lipid metabolism. *MEF2C*-deficient NK cells exhibit reduced expression of lipid biosynthetic enzymes such as stearoyl-CoA desaturase, impaired lipid storage, and diminished cytotoxic activity, defects that can be rescued by oleic acid supplementation [99].

**Fig. 3:**
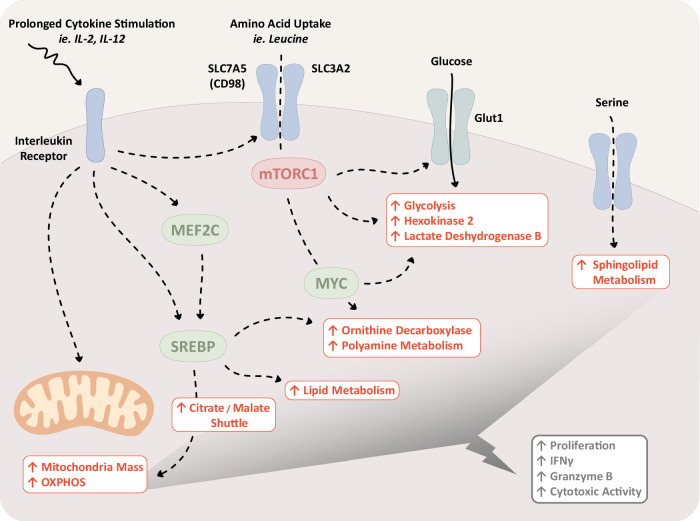
Crosstalk between signaling and metabolic pathways in NK cell activation. Prolonged cytokine stimulation engages central protein mediators—mTORC1, MYC, SREBP, and MEF2C—that act as hubs coordinating NK cell metabolic programs. These pathways integrate nutrient and cytokine cues to drive glycolysis, mitochondrial biogenesis, and the citrate/malate shuttle enabling the increase of mitochondrial oxidative phosphorylation (OXPHOS), while regulating amino acid-dependent pathways (i.e., leucine, arginine) to sustain mTORC1 activity, lipid biosynthesis, and polyamine production. Serine uptake supports one-carbon metabolism and sphingolipid biosynthesis. Together, this network enables NK cell proliferation, IFNγ and granzyme B production, and cytotoxic function.

Taken together, NK cells emerge as metabolically adaptable lymphocytes whose effector responses are tightly governed by nutrient availability, cytokine signaling, and transcriptional networks. Their reliance on non-canonical metabolic pathways such as the citrate/malate shuttle, their sensitivity to serine and amino acid availability, and the central integration of mTORC1 with SREBP, MYC, and MEF2C highlight metabolism as a defining regulator of NK cell biology (Fig. 3).

### Indirect metabolic regulation of T cell programs through epigenetic reprogramming

Metabolic reprogramming in lymphocytes during activation and differentiation not only meets energetic and biosynthetic demands but also generates metabolites that act as substrates or cofactors for chromatin-modifying enzymes, thereby linking metabolism to epigenetic regulation. While the metabolically-driven rewiring of the epigenome remains poorly understood in NK cells, T cells provide a clearer example, with key metabolic pathways directly controlling chromatin accessibility and transcriptional programs during activation and differentiation. Many of these pathways converge on H3 histone methylation or acetylation, in which one to three methyl groups or a single acetyl group are added to specific lysine residues to activate or repress gene expression in a context-dependent manner. For instance, H3K4me3 marks actively transcribed chromatin, whereas H3K27me3 is associated with repressed regions.

#### Nuclear acetyl-CoA affects histone acetylation in T cells

**Fig. 4:**
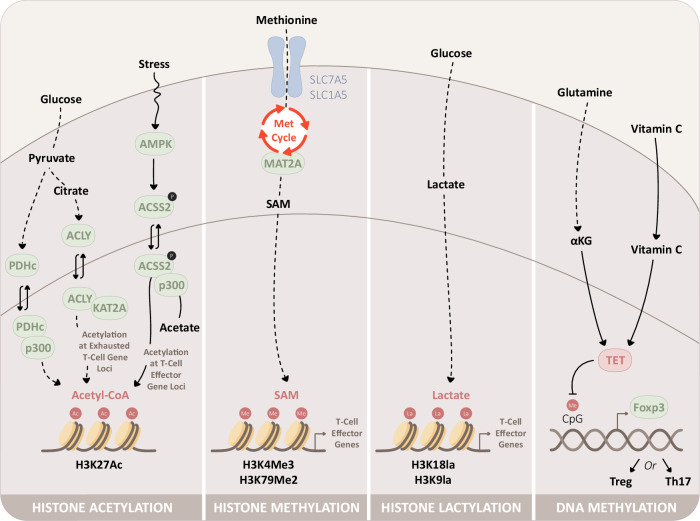
Impact of metabolism on the epigenetic rewiring of T cells. Critical metabolites serve as substrates or cofactors for chromatin-modifying enzymes, reshaping the T cell epigenetic landscape to promote effector gene expression. Nuclear acetyl-CoA, produced by ACLY, PDHc, or ACSS2, drives histone acetylation, enhancing chromatin accessibility and transcription of effector genes. Methionine metabolism supports S-adenosyl methionine (SAM) production via the methionine (Met) cycle, fueling histone methylation (i.e., H3K4me3, H3K79me2) and transcription of Th1/Th17-associated genes. Glycolysis-derived lactate promotes histone lactylation (i.e., H3K18la, H3K9la) at key genomic loci, further enhancing effector gene expression. Moreover, αKG from glutaminolysis, together with vitamin C, regulates TET DNA demethylases, enabling CpG demethylation and fine-tuning expression of critical T cell transcription factors like Foxp3.

Beyond its canonical roles in the TCA cycle and lipid biosynthesis, **acetyl-CoA** serves as a key substrate for histone acetylation, thus promoting chromatin opening and regulating gene expression [100]. The nuclear and cytosolic pools of acetyl-CoA are functionally distinct [101]. Because acetyl-CoA cannot cross membranes, its nuclear availability relies on localized production by acetyl-CoA-generating enzymes such as ACLY, the PDHc, and the acetyl-CoA synthetase short-chain family member 2 (ACSS2). These enzymes can translocate into the nucleus in response to specific stimuli to sustain histone acetylation and open defined chromatin regions which regulate the transcription of key regulator genes involved in T cell effector functions [102, 103] (Fig. 4).

Under normal nutrient conditions, Th17 cell stimulation promotes the expression of the glucose transporter *GLUT3* and *ACLY*. GLUT3-mediated glucose uptake fuels aerobic glycolysis and citrate production, providing the substrate for ACLY to generate acetyl-CoA. In differentiating Th1 cells, both malate-aspartate shuttle and mitochondrial citrate export are required for acetyl-CoA production [104]. This acetyl-CoA drives locus-specific histone acetylation, promoting inflammatory cytokine expression and supporting Th17 effector functions [105]. Consistent with this, deletion of *Acly* in murine CD8+ T cells leads to reduced acetyl-CoA synthesis, accompanied by decreased H3K27 acetylation and chromatin accessibility at specific genomic loci, including genes such as *Gzmb*, *Tbet*, *Cd44*, and *Ifng*, which are essential for CD8+ T cell effector function. In Th1 cells, impairing *Acly* or the mitochondrial citrate exporter *Slc25a1* similarly leads to decreased H3K9 acetylation [104]. These defects can be rescued by acetate supplementation, suggesting a metabolic interplay between the ACLY- and ACSS2-dependent pathways in driving T cell epigenetic rewiring upon activation [106]. Although nuclear translocation of ACLY has not been demonstrated in T cells, it has been observed in other cellular contexts to enhance histone acetylation [107], indicating that further studies are needed to determine how ACLY contributes to the nuclear acetyl-CoA pool in T cells.

In CD4+ T cells, early TCR engagement under nutrient-sufficient conditions promotes pyruvate accumulation, accompanied by the nuclear translocation of PDHc. PDHc has been reported to enter the nucleus either through direct interactions between clustered mitochondria and the nuclear envelope or via stepwise transport of individual subunits that are subsequently reassembled within the nucleus [108, 109]. In the nucleus, PDHc interacts with p300, a histone acetyltransferase, and with histones, facilitating nuclear acetyl-CoA synthesis required for acetylation of H2B, H3, and H4 histones, thereby regulating chromatin accessibility and epigenetic rewiring of CD4+ T cells [108, 110]. Consistent with these results, knockout of the pyruvate-to-lactate interconversion enzyme, *LDHA*, disrupts acetyl-CoA production and the epigenetic landscape in these cells [111].

Under low-glucose conditions, ACSS2 becomes critical in CD8+ T cells, where it converts acetate into acetyl-CoA to enhance histone acetylation and chromatin accessibility at specific genomic loci of key CD8+ regulator genes such as IFNγ in order to support optimal effector T cell function [112]. In non-T cell models, ACSS2 was shown to translocate into the nucleus during oxygen or serum limitation, recycling acetate generated locally by histone deacetylation back into acetyl-CoA [101]. During metabolic stress, such as under glucose starvation, this nuclear translocation is facilitated by AMPK-mediated phosphorylation of ACSS2 at serine 659, which exposes its nuclear localization signal and enables its interaction with the transcription factor TFEB [113]. Recent findings revealed that fluctuations in *ACSS2* expression are pivotal for CD8+ T cell fate. Downregulation of ACSS2 drives a metabolic shift from acetate to citrate utilization, favoring ACLY activity. This switch promotes citrate-dependent histone acetylation via ACLY-KAT2A interactions at exhaustion signature genes, while reducing acetate-dependent acetylation, mediated by a p300-ACSS2 complex, at effector and memory gene loci. Importantly, restoring nuclear ACSS2 or inhibiting ACLY prevents this CD8+ T cell exhaustion and enhances tumor-specific T cell responses [114].

#### Methionine metabolism regulates T cell histone methylation

Histone and DNA methylation depend on **S-adenosyl methionine** (SAM), the universal methyl donor. SAM is a key component of the methionine cycle and is produced from methionine by methionine adenosyltransferase enzymes such as MAT2A [115]. In activated CD4+ T cells, SAM biosynthesis depends on the import of extracellular methionine, driven by the upregulation of specific transporters such as *SLC7A5* and *SLC1A5* [116, 117]. Limiting methionine availability, or silencing *Mat2a* in mouse CD4+ T cells, reduces global H3K4me3 levels and the expression of Th17-associated genes, including *Il-17a* and *Batf* [117]. Similarly, methionine restriction in CD4+ and CD8+ T cells causes a loss of H3K79 dimethylation, leading to reduced expression of *Stat5* and *Ampk* respectively, and impaired T cell function [118, 119] (Fig. 4).

#### αKG levels influence T cell DNA methylation

In addition to acetylation and methylation, demethylation is a critical regulator of T-cell epigenetic programs. Demethylation occurs at both the histone level, mediated by JmjC-domain histone demethylases, and at the DNA level through removal of methyl groups from 5-methylcytosine (5mC). DNA methylation predominantly occurs at CpG sites and is dynamically regulated by **TET dioxygenases**, which catalyze the stepwise oxidation of 5mC to 5-hydroxymethylcytosine (5hmC), and subsequently to 5-formylcytosine and 5-carboxylcytosine [120]. These oxidized cytosine derivatives are then removed by base excision repair or diluted during DNA replication, resulting in active or passive DNA demethylation [121, 122].

Among the TET family members, *Tet2* and *Tet3* are more highly expressed than *Tet1* in mouse thymocytes and peripheral T cells [123]. Genome-wide analyzes show that 5hmC is enriched at regulatory elements of lineage-specific genes in distinct Th subsets, where Tet2 promotes transcription factor binding, including p300, and supports cytokine expression [124]. Accordingly, *Tet2* deletion reduces 5hmC levels and impairs Th1 and Th17 cytokine production in vitro and in vivo [124], while paradoxically enhancing memory CD4+ T cell expansion upon secondary viral challenge [125]. In CD8+ T cells, TET2 drives epigenetic programs favoring terminal differentiation at the expense of stem-like progenitor states [126]. These key functions have prompted the development of adoptive T-cell therapy strategies that exploit *TET2* disruption to enhance antitumor efficacy [126, 127].

The enzymatic activity of TET enzymes depends on metabolic cofactors, in particular **vitamin C** and **αKG**. In CD4+ T cells, vitamin C enhances demethylation of CpG motifs within the *Foxp3* intronic enhancer in a Tet2-dependent manner, promoting expression of *Foxp3*, the master transcription factor for Treg lineage specification, and driving Treg differentiation [128, 129]. Similarly, αKG, primarily generated from glutamine through glutaminolysis—where glutamine is first converted to glutamate by glutaminase and then to αKG via glutamate dehydrogenase or transaminases—serves both as a TCA cycle substrate to support biosynthetic processes and as a critical cofactor for TET demethylases. Consistent with the central role of glutamine in promoting CD4+ T cell differentiation into Th1 and Th17 cells (as described earlier in the amino acid section), glutamine starvation depletes intracellular αKG, reducing DNA methylation at the *Foxp3* locus and thereby increasing *Foxp3* expression, which skews differentiation toward Treg cells. Conversely, elevated αKG levels can drive Treg cells toward a Th1-like pro-inflammatory phenotype, characterized by hypermethylation of the *Foxp3* locus, decreased *Foxp3* transcription, and increased secretion of IFNγ, IL-17A, TNFα, and GM-CSF [56, 130] (Fig. 4).

In contrast, certain metabolites function as competitive inhibitors of αKG-dependent demethylases. For instance, an increased **succinate-to-αKG** ratio resulting from knockout of the mitochondrial complex II subunit *Sdhb* in CD4+ T cells enhances chromatin accessibility and promotes transcription of pro-inflammatory genes, driving the CD4+ T cell transition toward an inflammatory phenotype [131]. Similarly, the TCA cycle-derived metabolite S-2-hydroxyglutarate (S-2HG) inhibits αKG-dependent demethylases in CD8⁺ T cells and thereby modulates their differentiation [132]. Exogenous S-2HG supplementation alters global methylation patterns of both the H3K27 histone mark and DNA, closely mirroring the effects observed upon knockdown of the histone demethylase *KDM6A* and the DNA demethylase *TET2*, respectively [132]. Similarly, enzymatic demethylation of H3K27me3 by KDM6A and KDM6B is required for proper differentiation of CD4+ T cells from DP thymocytes [133]. Collectively, these findings underscore the central role of metabolic regulation of histone and DNA demethylases, such as TET2 and KDM6A/B, in driving the epigenetic reprogramming necessary for T lymphocyte differentiation.

#### Emerging role of other histone modifications on T cell function

Beyond canonical acetylation and methylation, novel histone modifications such as lactylation, crotonylation, propionylation, and butyrylation directly link metabolite availability to chromatin remodeling. Lactate-derived histone **lactylation**, for example, acts as an epigenetic mark that stimulates gene transcription [134]. In activated human and murine CD8+ T cells, increased glycolysis elevates lactylation of H3K18 and H3K9 at *Stat1*, *Cd28*, *Tcf7*, *Ccr7*, and *Batf3* gene loci, which are critical for T cell activation, an effect that can be blocked by small-molecule inhibitors of LDHA [135]. Glycolysis also influences epigenetic remodeling via lysine acetyltransferase 6A (KAT6A), which coordinates glucose metabolism with histone acetylation at glycolytic gene loci to support optimal CD4 + T cell responses [136].

In summary, both canonical modifications, such as histone acetylation and methylation, and newly described modifications, such as lactylation, integrate metabolic state with transcriptional programs to shape T cell differentiation, effector function, and memory formation (Fig. 4).

## Metabolic regulation of macrophages

### Direct metabolic regulation of macrophages differentiation and function

Macrophages are highly adaptable innate immune cells that adjust their phenotype in response to environmental cues, allowing them to balance antimicrobial defense and tissue repair with associated inflammation and tissue damage. In vivo, macrophage activation reflects a combination of monocyte-to-macrophage developmental pathways, tissue-specific cues that vary across organs and local inflammatory conditions, and polarizing signals provided by cytokines and modulated by metabolites [137].

A simplified framework has divided macrophages into two polarized states that can be reproducibly induced in vitro: classically-activated pro-inflammatory M1 macrophages, which are induced by Toll-like receptor (TLR) agonists such as lipopolysaccharide and IFNγ, and alternatively-activated anti-inflammatory M2 macrophages, induced by IL-4 and IL-13 [138]. More recently, additional subcategories of M2 polarization were reported. These comprise M2b, which arises in response to immune complexes and IL-1 receptor signaling and plays a regulatory role in immune and adaptive responses, and M2c, driven by IL-10, TGFβ, and glucocorticoids, contributing to immune suppression and inflammation resolution. Although this M1/M2 dichotomy does not capture the full complexity of macrophage states in vivo, it has provided a compelling model to explore how metabolism integrates immune signals to shape macrophage function.

**Fig. 5:**
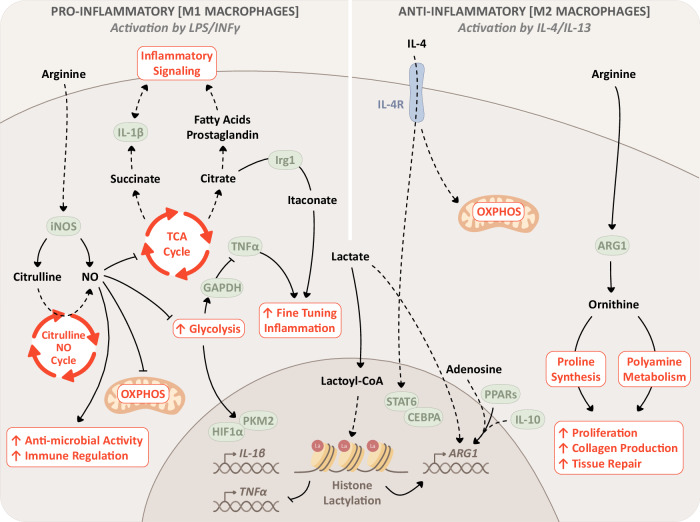
Arginine metabolism is a central determinant of macrophage polarization. In pro-inflammatory M1 macrophages, arginine is metabolized by iNOS into nitric oxide (NO) and citrulline, supporting antimicrobial activity, glycolysis, a disrupted TCA cycle, citrate/succinate accumulation, ROS production, fatty acid synthesis, and pro-inflammatory cytokine expression (i.e., IL-1β, TNFα). In anti-inflammatory M2 macrophages, arginine is metabolized by ARG1 into ornithine, fueling polyamine and proline synthesis for tissue repair and modulating local T cell responses. M2 polarization depends on oxidative phosphorylation fueled by fatty acid oxidation, with additional regulation via epigenetic mechanisms (acetyl-CoA, lactate, SAM) and post-transcriptional pathways that balance inflammation and tissue homeostasis.

One of the earliest hallmarks distinguishing M1 and M2 macrophages is their divergent use of arginine. IFNγ-activated M1 macrophages express inducible **nitric oxide synthase** (*iNOS*), which metabolizes arginine into nitric oxide (NO) and citrulline [139]. NO is critical for microbial killing and immune regulation [140]. To sustain its production, M1 macrophages upregulate cationic amino acid transporters such as SLC7A2 [141] and recycle citrulline through the citrulline-NO cycle, thereby maintaining NO synthesis when extracellular arginine becomes limiting (Fig. 5).

By contrast, IL4-induced M2 macrophages have negligible iNOS expression, which is regulated by IFNγ, but they display high expression of **arginase 1** (*ARG1*), which diverts arginine into ornithine (a precursor for polyamine metabolism) and urea. *ARG1* expression is driven by the transcription factor STAT6, which, upon IL-4 activation, binds an *ARG1* enhancer in cooperation with C/EBPβ [142, 143]. *ARG1* can also be upregulated by IL-10 via increased IL-4 receptor expression, by TGFβ, or through hypoxia, lactate, adenosine, and PPAR pathways [144–148].

Arginine consumption by ARG1 has two main effects. First, ornithine supports polyamine metabolism for cell proliferation and proline synthesis for collagen production and tissue repair. Second, macrophage ARG1 modulates T cell responses in a paracrine manner by locally depleting extracellular arginine, which, as described above, is essential for T cell proliferation and activity. This effect seems to be organ-specific as macrophage-specific deletion of *Arg1* during *Schistosoma mansoni* infection leads to uncontrolled, T cell-driven inflammation predominantly in the liver, suggesting that ARG1 activity in M2 macrophages fine-tunes T cell responses, particularly in organs with limited extracellular arginine availability [149]. Thus, arginine metabolism not only distinguishes macrophage subsets but could also affect the function of surrounding immune cells.

Differences in the anabolic use of arginine by macrophages also extend to central energy metabolism. NO poisons the respiratory chain of mitochondria, forcing activated M1 macrophages to import glucose and create energy from **glycolysis** [150]. Glycolysis provides rapid ATP, supports biosynthesis through the pentose phosphate pathway, and generates NADPH for the production of ROS by NADPH oxidase. TCA cycle is disrupted, leading to citrate accumulation, which supports fatty acid and prostaglandin synthesis, and succinate accumulation, which stabilizes HIF1α and promotes pro-inflammatory IL-1β production [150, 151]. Downstream of citrate and acetyl-CoA, fatty acid synthesis is an integral part of the M1 macrophage’s inflammatory program and organizes the plasma membrane for inflammatory signaling [152]. Additional glycolysis-related enzymes and metabolites are also involved in controlling the M1 macrophage inflammatory program: pyruvate kinase M2 translocates to the nucleus to promote HIF1α-dependent transcription of *IL-1β* [153]; citrate can be converted to itaconate by *Irg1*, which inhibits succinate dehydrogenase and tempers excessive inflammatory signaling [154]; and finally, GAPDH can bind to *TNFα* mRNA when glycolytic flux is low, repressing its translation and subsequent production, a mechanism that mirrors its role in T cells mentioned above, where it binds *Ifng* mRNA when glycolysis is limited [151].

By contrast, IL4-induced M2 macrophages favor **mitochondrial metabolism**. Although IL-4 signaling via AKT and mTORC1 transiently increases glycolysis, which is needed for early induction of *Arg1* and other markers, long-term activation depends on OXPHOS fueled by FAO [155, 156]. Lipid uptake through CD36 and mitochondrial import via CPT1A support this oxidative program [155]. While FAO was initially considered purely anti-inflammatory, it also contributes to inflammasome activation by oxidizing palmitate to generate mitochondrial ROS and activate NLRP3 [157]. Consistent with these findings, saturated long-chain fatty acids, particularly stearate and palmitate, were recently shown to increase expression of inflammasome priming genes such as *Nlrp3* and *Il-1β* in mouse macrophages and to induce secretion of mature Il-1β [158]. In conclusion, macrophage polarization is actively shaped by the differential use of arginine metabolism and of glycolytic versus OXPHOS pathways, whose distinct anabolic outputs drive alternative polarization fates and, consequently, either inflammatory or tissue-reparative programs (Fig. 5).

### Indirect metabolic regulation of macrophages differentiation and function

The **methionine cycle** integrates glycolysis and serine metabolism cues to support epigenetic rewiring and the activation of a pro-inflammatory program in M1 macrophages upon LPS stimulation. This occurs through increased production of SAM, which elevates H3K36me3 levels and enhances *IL-1β* expression [159]. Resolution of this inflammatory phenotype involves lactate, which, as in T cells, participates in chromatin remodeling and activates M2-like transcriptional programs in macrophages. During late M1 polarization, lactate accumulation initiates a “lactate clock,” whereby lactoyl-CoA—derived from extracellular or intracellular lactate—is incorporated into histones via the p300/p53 complex. This modification represses M1-associated genes, such as *TNFα*, while inducing M2-associated homeostatic genes, such as *ARG1*, facilitating the transition from a pro-inflammatory M1 phenotype to an anti-inflammatory M2 state [134]. Acetyl-CoA production via ACLY also contributes to this M1-to-M2 transition by enhancing histone acetylation [160, 161].

Beyond histone modifications, regulation of metabolism-related gene expression at the post-transcriptional level also shapes macrophage function. For example, ribosomal RNA processing 1 (RRP1) binds nuclear thymidylate synthetase (*TYMS)* transcripts and reduces its expression in inflammatory macrophages, thereby suppressing folate cycle activity and dampening one-carbon metabolism-driven inflammation [162]. Together, these findings highlight how metabolic intermediates, either through metabolite-driven chromatin modifications or RNA stability control can indirectly regulate macrophage function.

## Impact of metabolic intervention on immunotherapies

The metabolic pathways outlined above, which encompass energy production, amino acid utilization, lipid metabolism, and ion-dependent signaling, are essential for T cell, NK cell, and macrophage differentiation, proliferation, and effector function. In a general context, immune cells obtain these key metabolites from the extracellular milieu; in cancer, however, tumor metabolic reprogramming, which includes enhanced glycolysis, glutamine addiction, and increased fatty acid synthesis, creates competition for nutrients within the TME. In addition, tumor-derived metabolites, such as lactate, acidify the TME and further suppress immune activity, impairing, for instance, T and NK cell activation, and promoting M2-like macrophage polarization [15, 26, 163–165]. This metabolic competition dampens the efficacy of immune-based therapies such as adoptive cell therapies and antibody-mediated checkpoint blockade. Recognizing this tug-of-war, recent strategies target dietary modulation or key metabolic enzymes to restore nutrient availability, relieve metabolic stress, and enhance anti-tumor immune responses.

### Impact of metabolic intervention on immune checkpoint inhibitors

The efficacy of ICIs depends on the presence and functional competence of cytotoxic tumor-infiltrating T lymphocytes (TILs) within the TME, as the density of TILs is one of the strongest predictors of response to anti-PD-1/PD-L1 therapies [166]. Because T cells rely on tightly regulated metabolic programs to sustain activation, proliferation, and cytotoxicity, metabolic constraints imposed by tumors can severely impair their activity, prompting metabolic strategies to restore T cell fitness and potentiate ICIs efficacy.

### Impact of energy metabolism modulation on ICI response

A major area of investigation in enhancing ICIs efficacy has focused on tumor and immune cell **glycolysis** and **lactate dynamics**. Tumor cells exhibit a preference for aerobic glycolysis, leading to elevated lactate secretion that acidifies the TME and promotes immune evasion. As mentioned earlier, lactate was reported to drive histone H3K18 lactylation, resulting in upregulation of *PD-L1* and suppression of CD8+ T cell cytotoxicity; pharmacological inhibition of this pathway enhances the therapeutic efficacy of PD-1 blockade [167]. In parallel, targeting monocarboxylate transporters (MCT1 and MCT4), which mediate lactate export, using small-molecule inhibitors such as VB124 and diclofenac, mitigates TME acidification, sustains T cell activation, and enhances responsiveness to anti-PD-1 therapy [168, 169]. CD8+ TILs are also prone to accumulating mitochondria with compromised mitochondrial membrane potential in the TME. Supplementation with nicotinamide riboside helps prevent mitochondrial dysfunction and improves responsiveness to PD-1 blockade treatment [170].

**Glucose availability** also represents a key metabolic checkpoint within the TME. Under conditions of low glucose and oxygen in the TME, CD8+ T cells shift towards FAO for energy production. Pharmacological activation of FAO using peroxisome proliferator-activated receptor-α (PPARα) agonists such as fenofibrate was shown to synergize with PD-1 blockade, promoting T cell survival and delaying tumor progression [171]. Beyond its role in energy metabolism, intermediary metabolites of the TCA cycle can also directly modulate immune checkpoint pathways. For example, succinyl-CoA mediates succinylation of PD-L1 at lysine 129, promoting its degradation. Upregulation of *CPT1A* by bezafibrate enhances this process, synergizing with CTLA-4 blockade [172].

**Fig. 6:**
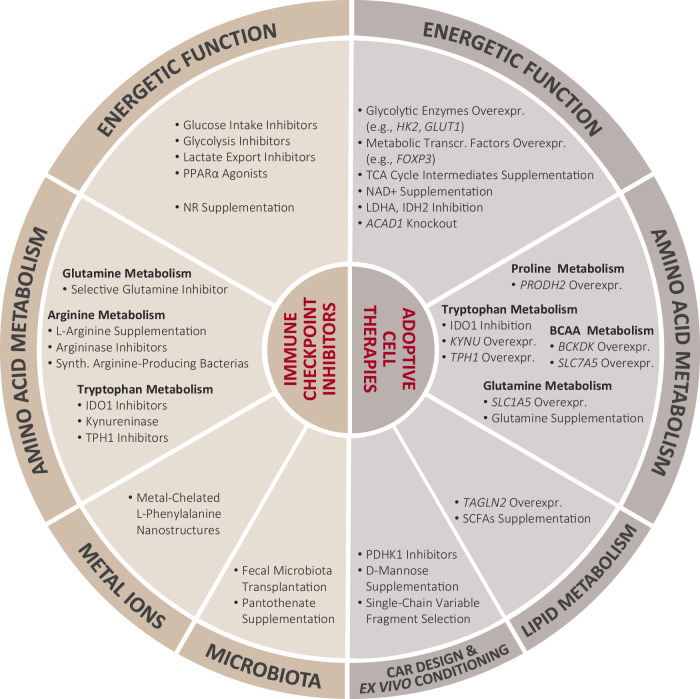
Metabolic interventions to optimize antitumor immunotherapies. Strategies include modulation of energy, amino acid, lipid, and metal ion metabolisms to enhance immune checkpoint inhibitors and CAR T cell efficacy. Emerging approaches such as fecal microbiota transplantation and metabolic priming of CAR T cells further expand this therapeutic arsenal. NR = Nicotinamide Riboside.

Efforts to selectively disrupt tumor glycolysis without impairing T cell metabolism have also shown promise. Tumor cells predominantly express *GLUT1*, while T cells also utilize *GLUT3*, to transport glucose. Inhibition of GLUT1 with the selective inhibitor BAY-876 spares T cell glucose uptake, enhances immune infiltration, and achieves durable tumor control when combined with PD-1 blockade, in particular in sustained-release pharmacological formulations [173, 174] (Fig. 6).

### Enhancing amino acid metabolism in TILs

As described in the earlier T cell metabolism section, **tryptophan catabolism** generates kynurenine via IDO1, which suppresses T cell function in a paracrine manner. Combining IDO1 inhibitors or extracellular kynurenine-degrading enzymes, such as PEGylated kynureninase, with PD-1 or CTLA-4 blockade outperforms the effects of each of these ICIs administered alone [175, 176]. Serotonin, another tryptophan derivative, similarly dampens CD8+ T cell activity; pharmacological serotonin depletion using fluoxetine or TPH1 inhibitors enhances PD-1 blockade and long-term tumor control [177].

**Arginine** is also a critical determinant of effective T and NK cell effector functions. In the TME, arginine is often depleted by tumor-expressed arginase as well as by myeloid-derived suppressor cells and polymorphonuclear cells, limiting T cell proliferation and anti-tumor immunity. Pharmacological inhibition of extracellular **arginase** with agents such as CB-1158 or AZD001 can restore T cell function, reduce tumor growth, and potentiate PD-1/PD-L1 or NK checkpoint inhibitors [178, 179]. Similarly, systemic **L-arginine supplementation** enhances T cell proliferation, differentiation, and survival, expanding tumor-specific CD8+ T cells and improving anti-PD-1/PD-L1 efficacy in preclinical models of osteosarcoma and colon carcinoma [180, 181]. Another synthetic biology approach, using an engineered probiotic Escherichia coli Nissle 1917 strain that colonizes tumors and converts accumulated ammonia into L-arginine, offers an alternative strategy to locally boost intratumoral L-arginine levels. This local increase in arginine promotes T cell infiltration and synergizes with PD-L1 blockade to enhance tumor clearance [182]. Beyond arginine metabolism, dietary interventions such as a **serine/glycine**-restricted diet can also enhance anti-tumor immunity by increasing CCL5 and CXCL11 secretion, thereby promoting CD8 + T cell recruitment and activation and potentiating anti-PD-1 therapy in preclinical and early-phase clinical studies [183].

Finally, **glutamine metabolism** represents another major axis of regulation. Tumor cells compete with T cells for glutamine, critical for proliferation and TCA cycle anaplerosis. While glutaminase inhibition alone shows limited efficacy, broad glutamine blockade with 6-diazo-5-oxo-L-norleucine (DON) or its TME-activated prodrugs inhibits tumor growth while conditioning CD8+ TILs toward a proliferative, long-lived, and highly activated phenotype [184]. These interventions relieve glutamine restriction on T cells and synergize with PD-1/PD-L1 therapy, although careful agent selection is required to minimize toxicity and preserve effector T cell metabolism (Fig. 6).

### Development of metal ions-based systems to modulate TILs signaling

**Metal ions** are critical regulators of immune signaling, and modulation of ion fluxes has emerged as a strategy to enhance antitumor immunity. A recent work demonstrated that metal-ion-chelating L-phenylalanine (L-Phe) nanostructures formed by coordination of Mg²⁺, Fe²⁺, or Zn²⁺ with L-Phe can disassemble within lysosomes to release functional complexes. These metal-chelated L-Phe dimers activate Kv1.3 potassium channels, triggering K⁺ efflux and Ca²⁺ influx, which in turn activate the calmodulin-dependent NF-κB pathway. This cascade enhances CD8+ T cell infiltration and potentiates the efficacy of anti-PD-1 therapy [185].

### Systemic influence of the microbiome on response to ICIs

The gut microbiome plays a pivotal role in shaping responses to ICIs by modulating both systemic and intratumoral immunity. A “favorable” microbiome — enriched in taxa such as *Ruminococcaceae* and *Faecalibacterium* — enhances antigen presentation and supports CD8+ T cell function, whereas an “unfavorable” microbiome, characterized by low diversity and increased abundance of Bacteroidales, is associated with impaired immune infiltration and reduced antigen presentation, correlating with poor responses to PD-1 blockade [186]. **Fecal microbiota transplantation**, in both clinical and preclinical settings, was reported to restore CD8+ T cell activity and reduce immunosuppressive IL-8-producing myeloid populations, confirming the causal role of microbiota composition in modulating ICIs efficacy [187].

While many aspects of microbiota composition can influence responses to ICIs, the role of microbial metabolite production is still being actively explored. Among these metabolites, vitamin B5 (pantothenate) has emerged as a key immunomodulator. Although diet is the primary source of **vitamin B5**, the gut microbiome also produces it, contributing to the host’s systemic pool. As a precursor of coenzyme A (CoA), vitamin B5 links amino acid catabolism, glycolysis, and FAO, and provides acetyl groups essential for histone acetylation. In mouse models of colon carcinoma, pantothenate supplementation enhances the efficacy of PD-L1 blockade, while in melanoma patients, higher baseline plasma levels of pantothenate are associated with improved responses to anti-PD-1 therapy [188] (Fig. 6).

### Impact of metabolic intervention on adoptive cell therapies

TME-associated metabolic stress also impacts the persistence and memory formation of infiltrating CAR cells. As with ICIs, tumor-driven metabolic constraints limit the ability of adoptive cell therapies to mount effective antitumor responses. Current research focuses on enhancing the function of these therapies either through the genetic engineering of CAR cells to resist TME-imposed metabolic barriers or by metabolically priming them to maximize their antitumor activity.

### Engineering CAR cells to overcome amino acid metabolic barriers in the TME

Multiple strategies have been developed to engineer CAR-T cells that can overcome amino acid-driven immunosuppression in the TME. Initial efforts focused on mitigating the inhibitory effects of **tryptophan metabolism** on CAR-T cell functions. CAR-T cells were modified to resist kynurenine-mediated functional impairment through pharmacological or genetic inhibition of the kynurenine-producing enzyme IDO1, which reduced CAR-T cell exhaustion and enhanced antitumor efficacy in gastric and pancreatic adenocarcinoma models [189]. Similarly, overexpression of the L-kynurenine hydrolase *KYNU*, which degrades the immunosuppressive kynurenine, improved CAR-T cell expansion, memory differentiation, and tumor-killing capacity, even in kynurenine-rich environments [190]. Finally, CAR-T cells engineered to overexpress *TPH1*, the enzyme that drives serotonin synthesis from 5-hydroxytryptophan (5-HT), displayed robust tumor control through intracellular 5-HT accumulation and subsequent GAPDH serotonylation. This modification promotes cytoplasmic GAPDH localization, shifts CAR-T cell metabolism toward glycolysis, and enhances antitumor activity [191].

**Glutamine metabolism** also represents a key target to enhance CAR-T cell function. Overexpression of the glutamine transporter *SLC1A5* (*ASCT2*) reprograms CAR-T cell metabolic fitness by activating mTORC1 signaling and improving both basal oxygen consumption and glycolytic activity, thereby sustaining CAR-T cell persistence in vivo [192]. Pretreatment with glutamine further activates the mTOR-SREBP2 pathway, strengthens immune synapse formation, and enhances proliferation, tumor infiltration, and memory maintenance [193].

**BCAA metabolism** provides another avenue for CAR-T cell optimization. CAR-T cells overexpressing *BCKDK*, a key regulator of BCAA catabolism, exhibit enhanced central memory differentiation and higher circulating CAR-T cell levels [194]. Similarly, overexpression of the leucine transporter *SLC7A5* stimulates mTORC1-driven glycolysis and cytokine secretion, further augmenting antitumor activity [195].

Finally, reprogramming **proline metabolism** through knock-in of *PRODH2*, which catalyzes the first step in trans-4-hydroxy-L-proline catabolism, enhances CAR-T efficacy in preclinical models of breast cancer and B-cell leukemia [196]. Together, these strategies illustrate that targeted manipulation of amino acid metabolism can significantly improve CAR-T cell fitness, functional persistence, and therapeutic efficacy within the metabolically hostile TME.

### Optimizing the energetic function of CAR cells

**Energy metabolism** is a major determinant of CAR-T cell function, and strategies to enhance CAR-T cell efficacy have initially focused on increasing the uptake of glycolytic substrates. Overexpression of glycolytic enzymes such as *HK2* or the glucose transporter *GLUT1* is sufficient to enhance the glycolytic flux in CAR-T cells and results in improved tumor control [197–199]. Similarly, engineering CAR-T cells to express the fructose transporter *GLUT5* enables them to utilize fructose in glucose-limited tumor niches, further enhancing their effector function and antitumor activity [200]. Energetic rewiring can also be achieved through overexpression of the transcription factor regulating T cell metabolism, FoxP3, which shifts CAR-T cells from aerobic glycolysis and oxidative phosphorylation toward increased lipid metabolism, thereby promoting sustained antitumor activity in vivo [201]. Finally, an alternative approach using a half-life-extended IL-10/Fc fusion protein has been shown to directly reinvigorate terminally exhausted CD8+ TILs by promoting OXPHOS through IL-10R activation and to improve the therapeutic efficacy of adoptive T cell transfer therapies [202].

Conversely, targeted inhibition of specific glycolysis- and TCA cycle-related enzymes can also enhance the function of CAR cell systems. LDHA inhibition increases CAR-T cell antitumor efficacy, *ACAD1* knockout in pluripotent stem cell-derived CAR-macrophages promotes pro-inflammatory polarization and tumor-suppressive activity, and IDH2 inhibition favors memory T cell differentiation through epigenetic remodeling [203–205]. Supplementation of TCA cycle intermediates has also proven effective: for example, succinate supports central memory phenotypes, enhances mitochondrial respiration, and improves CAR-T cell persistence, while NAD⁺ supplementation further increases CAR-T cell tumor-killing potential [206, 207].

### Modulation of lipid metabolism in CAR-T cell function

In addition to amino acid and energy metabolism, **lipid metabolism** has emerged as a critical determinant of CAR-T cell efficacy. Mevalonate and cholesterol synthesis enzymes are essential for TIL accumulation by supporting adaptations to tissue residency, including dependence on non-sterol products of the mevalonate/cholesterol pathway [208]. TAGLN2, which was recently identified as a new mediator of lipid uptake by promoting the localization of the fatty acid transporter FABP5 to the surface of CD8+ T cells, was exogenously expressed in CAR-T cells. *TAGLN2* overexpression enabled CAR-T cells to overcome tumor-induced endoplasmic reticulum stress, enhancing their therapeutic efficacy in preclinical models of metastatic ovarian cancer [209]. Similarly, short-chain fatty acids (SCFAs) such as pentanoate and butyrate can enhance CAR-T cell antitumor activity through combined metabolic and epigenetic reprogramming. SCFA treatment activates mTOR and inhibits class I histone deacetylases, leading to increased expression of CD25, IFNγ, and TNFα, effector molecules, and significantly improves CAR-T cell function in preclinical melanoma and pancreatic cancer models [210].

### Ex vivo conditioning of CAR-T cells

Beyond genetic engineering, **metabolic priming during** in vitro **expansion** has emerged as a critical determinant of CAR-T cell efficacy. A major limitation of adoptive cell therapy is the inability of ex vivo-activated CAR-T cells to persist long-term after infusion, in contrast to endogenous T cells that can maintain memory and survival for years. Conditioning CAR-T cells with the PDHK1 inhibitor dichloroacetate during in vitro expansion redirects their energetic metabolism toward a more “in vivo-activated”-like state, thereby improving survival and engraftment after transfer [211]. Another report showed that enhancing mannose metabolism in T cells via D-mannose supplementation during ex vivo manufacturing promotes stem-like features through O-GlcNAc transferase (OGT)-mediated O-GlcNAcylation and stabilization of β-catenin, which preserves *Tcf7* expression and epigenetic stemness. This reprogramming supports sustained CAR-T cell expansion and improves tumor control in vivo [212]. Importantly, beyond metabolic priming during ex vivo manufacturing, a key consideration in CAR design is that the choice of single-chain variable fragment used to recognize the tumor antigen can itself influence CAR-T cell glycolysis, amino acid uptake, and nutrient utilization, even in the absence of antigen, thereby shaping the metabolic resilience and antitumor effectiveness of a given CAR system [213].

## Conclusion

Over the last decade, immunometabolism has emerged as a central determinant of both endogenous and adoptive anti-tumor immunity. T cells, NK cells, and macrophages integrate nutrient availability, energy flux, and metabolite signaling to fine-tune proliferation, effector function, and differentiation. In cancer, tumor-imposed metabolic constraints profoundly impair immune activity and limit the efficacy of the latest immune-based therapies. Interventions that restore metabolic fitness, such as modulation of glycolysis, amino acid and lipid metabolism, and metabolite-driven epigenetic programs, have shown robust enhancement of both checkpoint blockade and CAR-based therapies.

Looking ahead, the next generation of CAR systems will likely go beyond traditional antigen specificity, incorporating metabolic resilience as a core design principle. Engineering CAR cells to sense and adapt to nutrient-depleted, hypoxic, and immunosuppressive TMEs through optimized amino acid transport, mitochondrial fitness, and epigenetic programming offers a path toward durable persistence, enhanced memory formation, and superior anti-tumor efficacy. Integrating insights from immunometabolism with synthetic biology and ex vivo metabolic priming could redefine adoptive cell therapy, enabling the rational design of CAR platforms that thrive metabolically within tumors.
